# Prospects for developing an accurate diagnostic biomarker panel for low prevalence cancers

**DOI:** 10.1186/1742-4682-11-34

**Published:** 2014-08-05

**Authors:** Matthew A Firpo, Kenneth M Boucher, Sean J Mulvihill

**Affiliations:** 1 Department of Surgery, University of Utah School of Medicine and the Huntsman Cancer Institute, Salt Lake City, UT, USA; 2 Department of Oncological Sciences, University of Utah School of Medicine and the Huntsman Cancer Institute, Salt Lake City, UT, USA

**Keywords:** Low-prevalence cancer, Biomarker, Accuracy, Weak classifier, Strong classifier panel, Pancreatic cancer, False positive diagnoses

## Abstract

**Background:**

Early detection screening of asymptomatic populations for low prevalence cancers requires a highly specific test in order to limit the cost and anxiety produced by falsely positive identifications. Most solid cancers are a heterogeneous collection of diseases as they develop from various combinations of genetic lesions and epigenetic modifications. Therefore, it is unlikely that a single test will discriminate all cases of any particular cancer type. We propose a novel, intuitive biomarker panel design that accommodates disease heterogeneity by allowing for diverse biomarker selection that increases diagnostic accuracy.

**Methods:**

Using characteristics of nine pancreatic ductal adenocarcinoma (PDAC) biomarkers measured in human sera, we modeled the behavior of biomarker panels consisting of a sum of indicator variables representing a subset of biomarkers within a larger biomarker data set. We then chose a cutoff for the sum to force specificity to be high and delineated the number of biomarkers required for adequate sensitivity of PDAC in our panel design.

**Results:**

The model shows that a panel consisting of 40 non-correlated biomarkers characterized individually by 32% sensitivity at 95% specificity would require any 7 biomarkers to be above their respective thresholds and would result in a panel specificity and sensitivity of 99% each.

**Conclusions:**

A highly accurate blood-based diagnostic panel can be developed from a reasonable number of individual serum biomarkers that are relatively weak classifiers when used singly. A panel constructed as described is advantageous in that a high level of specificity can be forced, accomplishing a prerequisite for screening asymptomatic populations for low-prevalence cancers.

## Background

With an annual incidence of 4 cases per 10,000 people in the United States, pancreatic ductal adenocarcinoma (PDAC) is a rare disease, but has the highest mortality rate of any cancer [1]. A substantial determinant for the lethality of PDAC is the late presentation due to asymptomatic development of the disease. Earlier detection may improve outcomes by identifying the disease while still amenable to potentially curative intervention. As with other low prevalence cancers, screening for PDAC in asymptomatic populations will require a highly accurate screening test in order to avoid the expense and distress associated with a high number of falsely positive identifications.

**Figure 1:**
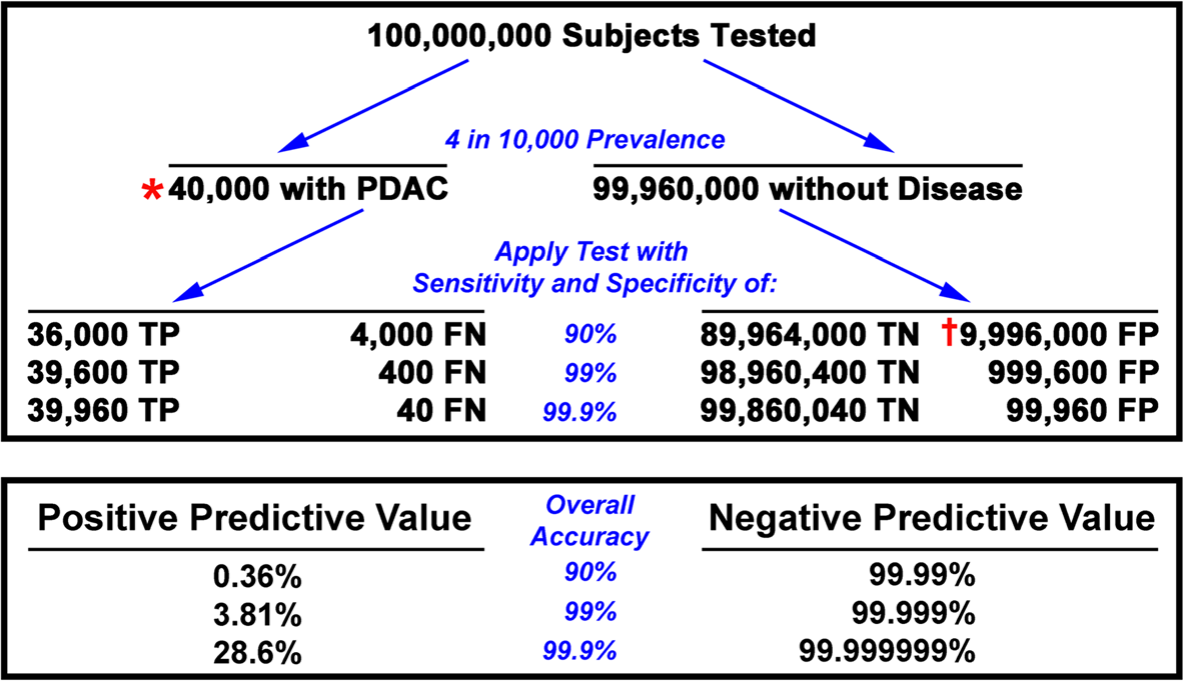
**Interpretation of test results at various levels of test accuracy for diagnosis of PDAC.** Outcomes are shown for a population of 100 million individuals (the current approximate number of individuals over the age of 50 in the United States) and assuming an annual disease prevalence of 4 in 10,000. FN = False-negative test results; FP = false-positive test results; TN = true-negative test results; TP = true-positive test results. Positive predictive value = probability of PDAC in a patient with a positive test result = TP/(TP + FP). Negative predictive value = probability of no PDAC in a patient with a negative test result = TN/(TN + FN). *$2.6 billion annual cost to treat [3]. †$5.5 billion annual cost for a single contrast-enhanced computed tomography (CT) follow-up screen for each false positive determination (based on 2011 Medicare technical and professional reimbursement rate of $554/CT).

Screening asymptomatic populations for cancers that have low prevalence presents challenges due to the potential for large numbers of falsely positive diagnoses. The problem is demonstrated in Figure 1, which illustrates a hypothetical screening scheme for people over the age of 50, a likely demographic for PDAC screening. In an asymptomatic United States population of about 100 million at risk individuals, about 40,000 will be discovered to have the disease annually [1]. Current biomarker tools (for example CA 19–9, [2]) can reach about 90% specificity, but screening at this level means that almost 10 million people will be falsely identified as having PDAC. Based on Medicare reimbursement rates, the cost of a single follow-up computed tomography (CT) scan on these false positive cases would be over $5 billion annually. This estimate does not include other costs such as clinic visits, blood tests, and further studies such as pancreatic endoscopic ultrasound evaluations that would be required to prove the absence of disease. Importantly, an additional barrier is the emotional distress of the individuals incorrectly diagnosed with a highly fatal disease. Contrasted with the cost of treating all true positive PDAC cases, which is estimated to be $2.6 billion annually in the US [3], the costs associated with false-negative identifications at the 90% assay specificity level is unsustainable. Increasing the specificity of the test would decrease the number of false positive evaluations to a more acceptable range [4]. Although open for debate, screening the asymptomatic population will likely require specificity greater than 99% to be practical.The trade-off between specificity and sensitivity means that specificity might be increased at the expense of sensitivity. However, in order for an assay to be meaningful for clinical management of the individual patient, high sensitivity must also be maintained. At current specificity levels of 90%, the probability of not having PDAC in a patient with a negative test result is very high (Figure 1). This high negative predictive value is a consequence of the rarity of the disease, with the ratio skewed by the large number of true negative cases. It follows that the positive predictive value, the probability of PDAC in a patient with a positive test result, is very low. Therefore, increasing sensitivity to 99% or greater is necessary in order for the individual test results to be clinically actionable.

Existing biomarkers, biomarker panels, and diagnostic algorithms fall well short of the accuracy levels required to bring the number of false-positive determinations in asymptomatic populations into an acceptable range [5-10]. Since PDAC develops from multiple different combinations of genetic and possibly epigenetic lesions [11,12], it seems logical that individual cancer cases may express a subset of markers while other cases express a different subset. Thus, attempts to identify a single test for discrimination of all PDAC cases may be frustrated because of disease heterogeneity. We developed mathematical models based on experimental data from nine serum biomarkers, all of which were significantly elevated in pancreatic cancer cases relative to controls. We asked if an accurate panel classifying tool could be developed from a group of these weak individual biomarkers and hypothesized that increased accuracy could be realized by allowing for multiple combinations of biomarkers, accommodating disease heterogeneity.

## Results

### Characteristics of individual PDAC biomarkers

**Figure 2:**
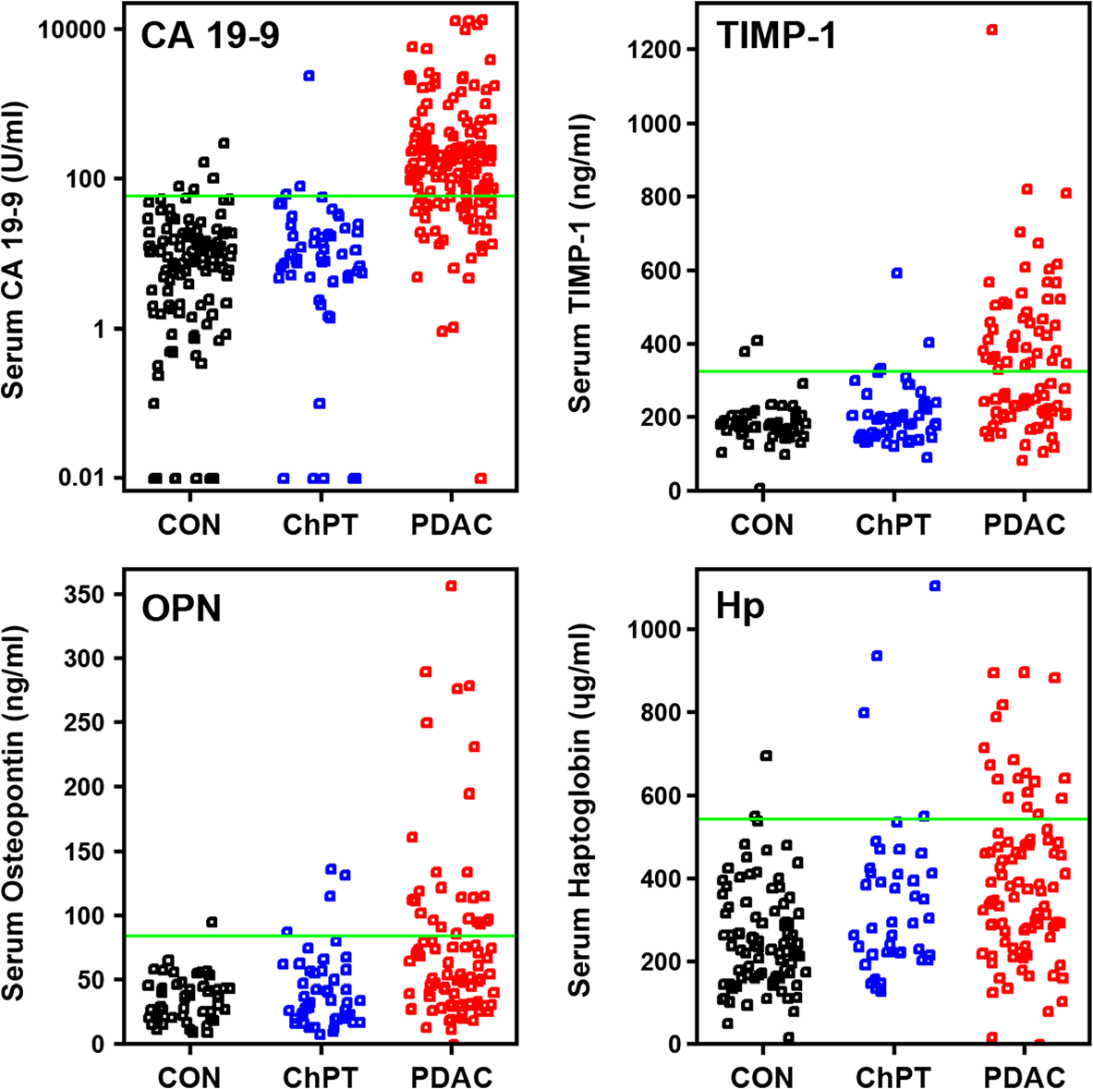
**Relative distributions for PDAC diagnostic biomarkers.** Levels of CA 19–9, haptoglobin (Hp), MMP-7, osteopontin (OPN), and TIMP-1 demonstrate considerable overlap in serum from healthy control subjects (CON), chronic pancreatitis patients (ChPT) and pancreatic ductal adenocarcinoma (PDAC) patients. Each data point represents the biomarker level for an individual sample. Note that serum CA 19–9 levels are presented using a logarithmic scale. Green horizontal lines indicate the 95% specificity threshold for the individual biomarkers.

To address the possibility of devising a test with 99% sensitivity and specificity, we sought to develop mathematical models based on experimental serum biomarker data. From previous experiments in which we determined levels of various biomarkers in serum from PDAC patients, chronic pancreatitis patients, and healthy subjects, we identified nine biomarkers whose mean levels were significantly elevated in PDAC cases relative to controls. These biomarkers were soluble AXL, CA 19–9, haptoglobin, soluble hyaluronic acid, matrix metallopeptidase 7 (MMP-7), matrix metallopeptidase 11 (MMP-11), osteopontin, serum amyloid A, and TIMP metallopeptidase inhibitor 1 (TIMP-1). Although the mean values of each of these biomarkers were significantly elevated in PDAC cases, accurate classification of individual results is problematic because of the large overlap of values within case and control groups. The individual biomarkers are thus weak diagnostic classifiers. The overlapping distributions for CA 19–9, haptoglobin, osteopontin, and TIMP-1 are shown in Figure 2. This observed overlap is consistent with disease heterogeneity in that individual cancer cases may develop to express a subset of markers while other cases express a different subset.

For the nine biomarkers, a sample set from 117 healthy control subjects, 58 chronic pancreatitis patients, and 159 PDAC patients was identified for which at least 3 of the 9 biomarkers were measured in individual samples. The median number of biomarkers queried per sample was 6 and missing data points were imputed. This final data set was used to identify biomarker characteristics for model development. To prioritize high specificity, we first assigned a diagnostic threshold (the indicator variable) at the 95th percentile of control values on the individual biomarkers and then calculated the resulting sensitivity. Between 17% and 75% of the PDAC cases had values above the 95% specificity threshold with an average sensitivity for all biomarkers of 32%.

Since direct correlation between biomarkers provides less diagnostic information than independent predictors, we also assessed the degree of correlation between the nine biomarkers within each group (PDAC, healthy controls, chronic pancreatitis). The correlation between the indicator variables was near zero in controls and slightly positive in PDAC cases. None of the biomarkers were highly correlated. The correlation in the PDAC samples had mean of 0.15 and median 0.13, but was highly variable (range −0.12 - + 0.44). The mean and median correlation in the controls was 0.12 and 0.088, respectively. Since the square of the correlation is the percentage of shared variation, markers shared about 2% of the variation in cases and 1-2% of variation in controls. This could be an overestimate, as missing data was imputed.

### Modeling PDAC biomarker panels

**Figure 3:**
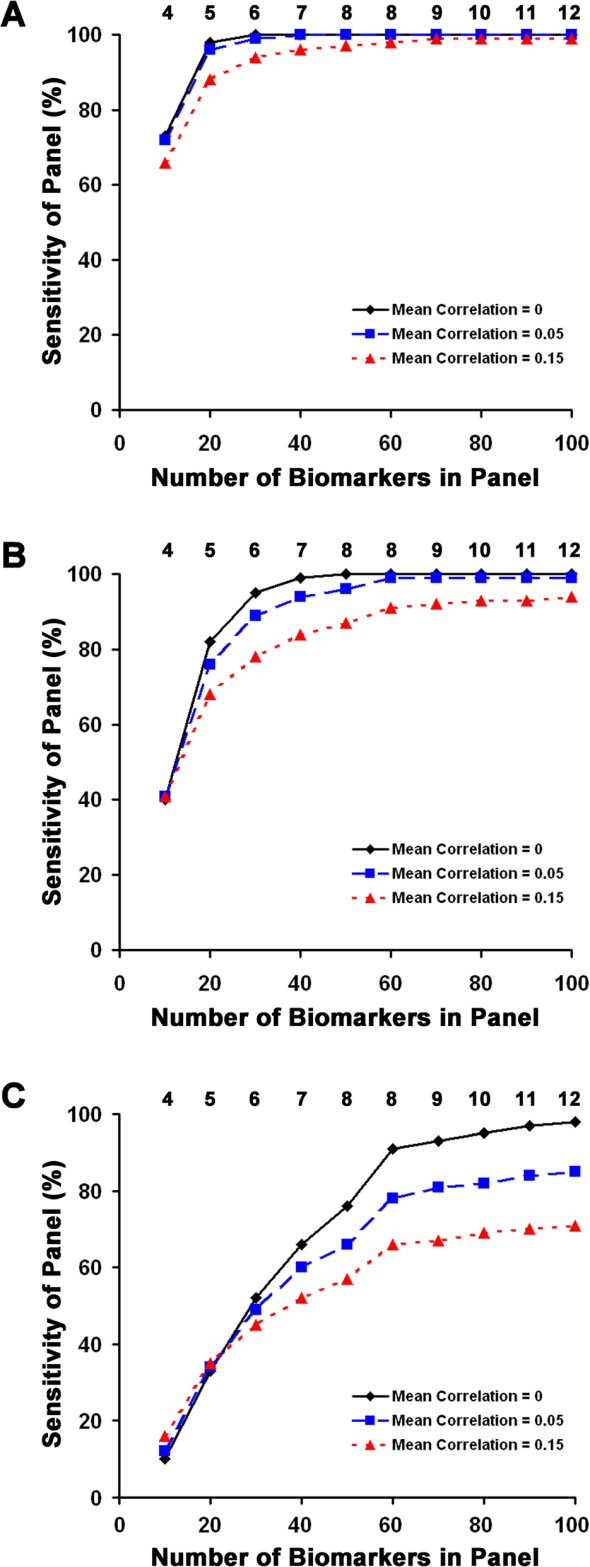
**Characteristics of biomarker panels with 99% specificity.** The modeled sensitivity of biomarker panels is given for panels consisting of between 10 and 100 individual biomarkers. The three panels represent different assumptions about the individual biomarkers: Chart **A** indicates panel characteristics assuming all biomarkers in the panel yield 45% sensitivity at the 95% specificity threshold (e.g. TIMP-1), Chart **B** indicates panel characteristics assuming all biomarkers in the panel yield 32% sensitivity at the 95% specificity threshold (the average of the 9 biomarkers examined), and chart **C** indicates panel characteristics assuming all biomarkers in the panel yield 19% sensitivity at the 95% specificity threshold (e.g. haptoglobin). Different assumptions about the mean correlation ratios between individual biomarkers constituting the panels are indicated by the color legend. Numbers above the data points indicate the minimum number of biomarkers in the panels required to be above the 95% specificity threshold in order to make a positive diagnosis of PDAC.

We modeled the behavior of a biomarker panel consisting of a sum of indicator variables, then chose a cutoff for the sum to force specificity to be high, and calculated the resulting sensitivity. To generate correlated biomarkers, we simulated correlated continuous biomarker data, made a 95th percentile cutoff for each biomarker and then assessed performance as above. The average correlation assumption was conservative in that we ignored inverse correlation in our modeling, which would tend to increase overall accuracy of the panel. Therefore, we also modeled the less conservative correlation assumption of 0.05.Modeling results for three panels that required 99% panel specificity, but were derived using different sensitivity assumptions about the individual biomarkers are shown in Figure 3. The model demonstrated, for example, that a panel consisting of 40 biomarkers characterized individually by 32% sensitivity at 95% specificity would require any 7 biomarkers to be above the threshold and would result in a panel sensitivity of at least 99% (Figure 3B), assuming no correlation between biomarkers. The addition of correlation assumptions reduced sensitivity for the 40-biomarker panel to 94% at an average correlation of 0.05 and 84% at an average correlation of 0.15. Increasing the mean sensitivity of the individual biomarkers from 19% to 45% in the panel not only reduced the number of biomarkers required for high accuracy, but also reduced the contribution of correlation between the individual biomarkers.

## Discussion

We asked if we could generate a “strong classifier” panel from a group of “weak classifiers”, with the stipulation that the algorithm allow for heterogeneity of the disease. The idea that a group of weak classifiers can be combined to form a strong-classifier is an established theoretical concept [13-15]. However, our goal of accommodating disease heterogeneity by allowing different biomarker subsets would increase the overall number of biomarkers necessary in the panel. Models developed using the characteristics of nine representative biomarkers measured in human samples revealed that panels with 99% specificity and sensitivity could be achieved using a reasonable number of biomarkers. For the purposes of modeling, the identity of the biomarkers is not critical since the only characteristic used in the central model (Figure 3B) was the average sensitivity at 95% specificity. The two other models (Figure 3A and C) provide information relevant to how different sensitivities arising from a different biomarker set might alter the number of total biomarkers required to achieve high accuracy. The nine biomarkers were chosen because they were significantly elevated in PDAC cases, providing credibility that they are related to the presence of disease. However, filtering biomarker data by insisting statistically significant differences between groups may mask potentially informative biomarkers in an analysis, such as ours, that allows diagnostic analyte subsets. Our approach is advantageous in that a high level of specificity can be forced and demonstrates that accommodating heterogeneity in the system has the potential to improve accuracy of cancer diagnostic biomarker panels, particularly for low-prevalence cancers.

Although our main goal was to evaluate if increased accuracy could be realized by allowing for disease heterogeneity, one limitation of our experimental design is that the dataset used biomarker levels from all PDAC stages. To be effective at improving outcomes, any diagnostic screening test should be able to identify early stage, treatable cases. Whether or not these biomarkers exist for PDAC will require further experimentation. The likelihood of finding 30–50 biomarkers with at least the average levels of accuracy seen in the nine biomarkers used here seems reasonable given that 162 secreted proteins are routinely over expressed in PDAC tumors [16] and other biomarkers, such as degraded cell-surface proteins, miRNAs, genetic mutations, and metabolic products could be incorporated to extend the panel. Since highly correlated biomarkers provide the same information, the most suitable biomarkers for inclusion in a panel will likely be those that identify different features of the disease. Finally, although increasing the accuracy of tests for low prevalence cancers would reduce the cost and distress associated with falsely positive determinations in screening of asymptomatic populations, an acceptable level for false-positive determinations is an open question that need be addressed by clinical discourse.

## Conclusions

Mathematical modeling of existing serum biomarker data indicates that, by allowing for diverse responses between cases, a biomarker panel can be devised that has greater than 99% accuracy for diagnosis of a low prevalence cancer, pancreatic ductal adenocarcinoma. Our results do suggest that limiting analysis to those biomarkers with only the highest accuracy may be counterproductive. The results provide a framework for identifying useful biomarker characteristics and minimizing biomarker correlation.

## Methods

### Ethics statement

All studies were carried out with the approval of the University of Utah Institutional Review Board and written informed consent was obtained for each participant enrolled in the study protocols.

### Serum biomarkers

Serum levels of AXL, CA 19–9, haptoglobin, hyaluronic acid, MMP-7, MMP-11, osteopontin, serum amyloid A, and TIMP-1 were measured in sera from 117 healthy control subjects and 58 chronic pancreatitis patients, and 159 PDAC patients collected prior to treatment. Control serum samples were obtained from healthy adults accompanying index patients to the Hunstman Cancer Institute Gastrointestinal Multidisciplinary Clinic. Diagnoses of PDAC cases were confirmed by histological evaluation and consisted of a range of stages (10 stage IA or IB, 20 stage IIA, 47 stage IIB, 30 stage III, and 52 stage IV). Diagnostic and prognostic characteristics for CA 19-9 [2], haptoglobin [6], osteopontin[17], serum amyloid A [6], and TIMP-1 [17] in our cohort have been previously published, as have prognostic characteristics for MMP-7 [18]. Biomarker characterization for AXL, hyaluronic acid, and MMP-11 will be published elsewhere. The median number of biomarkers queried per sample was 6. Missing data points (1103 of 3006 values) were imputed using the “aregImpute” function in the Hmisc package in R. A weighted multinomial probability sampling method with a tricube function as weights was used for imputation. The outcome variable was not used in imputation.

### Modeling

We modeled the behavior of a biomarker panel consisting of a sum of indicator variables, then chose a cutoff for the sum to force specificity to be high, and calculated the resulting sensitivity. To generate correlated biomarkers, we simulated independent normal random variables for each biomarker, and then added a common simulated random normal variable to each of them to introduce correlation. By varying the standard deviation of the common component, the correlation between the simulated biomarkers could be adjusted. We then made a 95th percentile cutoff for each simulated biomarker and assessed the performance as above. R statistical computing software version 2.8.0 (The R Foundation for Statistical Computing, Vienna Austria) was used for the simulations.

## Competing interests

The authors declare that they have no financial competing interests.

## Authors’ contributions

MAF conceived and implemented the study, performed data collection, participated in data analysis, and drafted the manuscript. KMB participated in the design of the study, performed the statistical analyses, and carried out the modeling. SJM participated in study design and conception and helped to draft the manuscript. All authors read and approved the final manuscript.
